# Gut-Microbiota-Driven Lipid Metabolism: Mechanisms and Applications in Swine Production

**DOI:** 10.3390/metabo15040248

**Published:** 2025-04-04

**Authors:** Shuqi Xiong

**Affiliations:** National Key Laboratory of Pig Genetic Improvement and Germplasm Innovation, College of Animal Science and Technology, Jiangxi Agricultural University, Nanchang 330045, China; chencongying@jxau.edu.cn; Tel.: +86-791-83813080

**Keywords:** gut microbiota, lipids, bacterial membranes, cholesterol, bile acids, choline, sphingolipids, fatty acids

## Abstract

**Background/Objectives:** The gut microbiota plays a pivotal role in host physiology through metabolite production, with lipids serving as essential biomolecules for cellular structure, metabolism, and signaling. This review aims to elucidate the interactions between gut microbiota and lipid metabolism and their implications for enhancing swine production. **Methods:** We systematically analyzed current literature on microbial lipid metabolism, focusing on mechanistic studies on microbiota–lipid interactions, key regulatory pathways in microbial lipid metabolism, and multi-omics evidence (metagenomic/metabolomic) from swine models. **Results:** This review outlines the structural and functional roles of lipids in bacterial membranes and examines the influence of gut microbiota on the metabolism of key lipid classes, including cholesterol, bile acids, choline, sphingolipids, and fatty acids. Additionally, we explore the potential applications of microbial lipid metabolism in enhancing swine production performance. **Conclusions:** Our analysis establishes a scientific framework for microbiota-based strategies to optimize lipid metabolism. The findings highlight potential interventions to improve livestock productivity through targeted manipulation of gut microbial communities.

## 1. Introduction

The gastrointestinal tract serves as a central hub for nutrient digestion, absorption, and metabolism and provides a dynamic interface for host–microbiota crosstalk. Members of the gut microbiota not only synthesize metabolites de novo but also convert dietary components and host metabolic products into bioactive compounds, particularly short-chain fatty acids (SCFAs), bile acids, and indole metabolites [1]. These metabolites act as critical mediators in microbiota–host physiological interactions. Emerging studies emphasize that integrating microbiome and metabolome data, rather than focusing solely on microbiota, offers deeper insights into microbiota-mediated physiological processes [2].

Lipids, broadly defined as hydrophobic biomolecules, are categorized into eight classes based on structural features, including Fatty acyls (FAs), Glycerolipids (GLs), Glycerophospholipids (GPs), Sphingolipids (SPs), Sterol Lipids (STs), Prenol Lipids (PRs), Saccharolipids (SLs), and Polyketides (PKs) [3]. Because of the complexity of lipid structure and biosynthesis, the current classification method for lipids is mostly based on their chemical compositions and hydrophobic and hydrophilic characteristics [4]. As of April 2025, over 49,667 unique lipid structures have been cataloged in the LIPID MAPS database (https://lipidmaps.org).

Beyond their roles as energy reservoirs and membrane components, lipids play important roles in regulating metabolic homeostasis [5] and have demonstrated connections to a variety of chronic illnesses, including metabolic syndrome components like obesity and diabetes, as well as cardiovascular pathologies [6]. Notably, gut microbiota profoundly influences lipid absorption, metabolism, and secretion. For instance, Bacteroidetes are associated with lean phenotypes, while specific Firmicutes taxa, such as *Faecalibacterium*, Lachnospiraceae, and Ruminococcaceae, show positive correlations with obesity [7]. Gut microbial diversity showed significant inverse relationships with serum triacylglycerols and BMI in humans [8]. Germ-free (GF) mice receiving fecal microbiota transplants obtained from twins with different obesity phenotypes acquired the obese phenotype from obese donors [9]. In addition, comparative analyses of conventionally raised (CONV-R) and GF mice demonstrated that the gut microbiota could significantly modulate lipid metabolism in serum, liver, and adipose tissue. For example, the levels of triglyceride and phosphatidylcholine were markedly altered [10]. GF mice exhibited impaired lipid absorption capacity in the duodenum and jejunum and had a relatively slower absorption rate of lipids across various organs compared to conventional mice [11]. A previous study highlighted the critical function of gastrointestinal microbial communities on lipid metabolism in livestock. For instance, pre-weaning supplementation with lipid mixtures and *Lactobacillus fermentum* in piglets was shown to selectively enrich *Clostridium* abundance in the gut, concurrently elevating cecal SCFA concentrations. This microbial shift not only enhanced intestinal secretory function but also contributed to metabolic homeostasis regulation [12].

Despite the significant advances in lipidome, the intricate crosstalk between lipids and gut microbiota, along with the mechanisms of lipid-mediated host–microbiota interactions remain poorly characterized. This review outlines the diversity and functional capacities of bacterial membrane lipids; describes the function of gut microbiota regulating the metabolisms of cholesterol, bile acids, choline, sphingolipids, and fatty acids; and discusses the effects on host physiology. Finally, we explored the potential application of lipid metabolites by gut microbiota in the improvement of production traits and health of livestock.

## 2. Bacterial Membrane Lipids

Bacterial membranes exhibit remarkable lipid diversity driven by adaptations to environmental stressors. Membranes are primarily formed by amphipathic lipids, with phospholipids as the dominant component. Although bacteria are considered to possess more limited types of lipids than plant or animal cells, they contain a surprising diversity of enzymes and the complex pathways for lipid synthesis. *Escherichia coli* has been considered as a standard model for studying bacterial membrane lipids with a relatively simple membrane composition. Approximately 75% of the phospholipid species present in *E. coli* cell membranes are phosphatidylethanolamine molecules, followed by phosphatidylglycerol (PG) and cardiolipin (CL) [13].

Phosphatidylcholine (PC), a neutral phospholipid critical for membrane integrity, accounts for ~50% of eukaryotic bilayers and is estimated to be found in approximately 15% of bacteria [14,15]. The amount of PC detected varies widely among bacterial species, constituting as little as 0–4% of total membrane lipids in *Pseudomonas aeruginosa* and reaching up to 73% in *Acetobacter aceti* [16,17]. Its electrical neutrality, rather than negative charge repulsion, is crucial for sustaining the structural integrity and functional properties of biomembranes [18]. PE constitutes a common membrane phospholipid in Proteobacteria and other Gram-negative bacteria [19]. Exhibiting a conical molecular structure, PE is primarily found in the inner plasma membrane bilayer and plays crucial roles in membrane curvature generation and fusion pore maintenance during exocytosis [20]. PE’s molecular architecture, characterized by a small hydrophilic headgroup and limited water affinity, facilitates tight intermolecular packing and confers exceptional stability at elevated temperatures [21]. These properties significantly enhance membrane rigidity and make PE an essential component in regulating membrane structure, permeability, and fluidity [22]. PG constitutes 20–25% of total phospholipid contents in most Gram-negative bacteria [23]. The homologous genes for synthesizing PG and its derivatives have been identified in genomes of various Proteobacteria species [19]. In the Gram-positive bacteria, PG occupied about 60% of total lipids [24]. Phosphatidylinositol (PI) widely exist in Actinomycetes and some δ-proteobacterial species [19]. PI and its metabolic derivatives, such as phosphatidylinositol mannose, linear and mature branched lipomannans, and lipoarabinomannan, are important components of the structure of bacterial cells and play crucial roles in bacterial physiological functions and the process of host infection in *Mycobacterium tuberculosis* [25]. Phosphatidylserine (PS), as an intermediate in PE biosynthesis, accumulates in only certain bacterial taxa. Typically, due to the exceptional efficiency of phosphatidylserine decarboxylase (Psd) in converting PS to PE, PS concentrations measured are often quite low [26]. PS and PI can determine the surface charge of the membrane and regulate interactions between lipid bilayers and cationic protein domains despite their low abundances [27].

Different bacteria show a distinct membrane lipid signature that is caused by both unique lifestyles and genetically encoded biosynthesis mechanism [28]. Studies on bacterial membrane phospholipid homeostasis have also focused on the adjustment of the structure in the glycerol backbone fatty acyl chains, including double bond count, position, and configuration, which directly affect biophysical properties of the membrane bilayer. Bacterial species have evolved sophisticated biochemical systems governing both the synthesis and architectural modification of fatty acids, enabling them to regulate membrane lipid composition and adapt to diverse physical environments [29]. For instance, CLA-HY, an enzyme identified in *Lactobacillus* and *Bifidobacterium*, can catalyze the conversion of linoleic acid to conjugated linoleic acid (CLA) [28]. Cis-trans isomerization of membrane phospholipids can result in higher phase transition temperatures, enhanced stiffness, and lower solute permeability of cell membranes [29]. In *Escherichia coli*, a decrease in temperature reduced membrane fluidity, which prompted a remodeling of cellular membranes characterized by elevated unsaturated fatty acid content. Conversely, bacteria growing in high-temperature environments typically made cell membranes enrich saturated fatty acids [30]. The fatty acyl components of bacterial membrane lipids which were added heterogeneous branches can influence the binding capacity and affinity of cellular receptors and fine-tune innate immunological perception [31,32].

Lipid components in bacterial cells serve several essential purposes: they ensure structural integrity of cellular membranes, enable energy production via respiratory chains, establish proper microenvironments for integral membrane proteins, and function as protective shields against hostile conditions. Therefore, understanding the structural characteristics and biological roles of bacterial membrane lipids is crucial for various applications, such as developing antibiotics targeting lipid biosynthesis or disrupting membrane integrity, ultimately contributing to novel strategies against bacterial infections. Although the integration of high-resolution lipidomic technologies and microscopic structural imaging has advanced our ability to comprehensively explore lipid profiles of bacterial membranes, it is still a challenge to fully characterize the distribution of lipids across diverse bacterial species. This complexity arises from the remarkable diversity of membrane lipids, which stems from variations in head group structures and acyl chain configurations, coupled with extensive biochemical modifications, including oxidation, reduction, conjugation, and substitution reactions [23].

## 3. Gut Microbiota Regulates Lipid Metabolism

**Figure 1:**
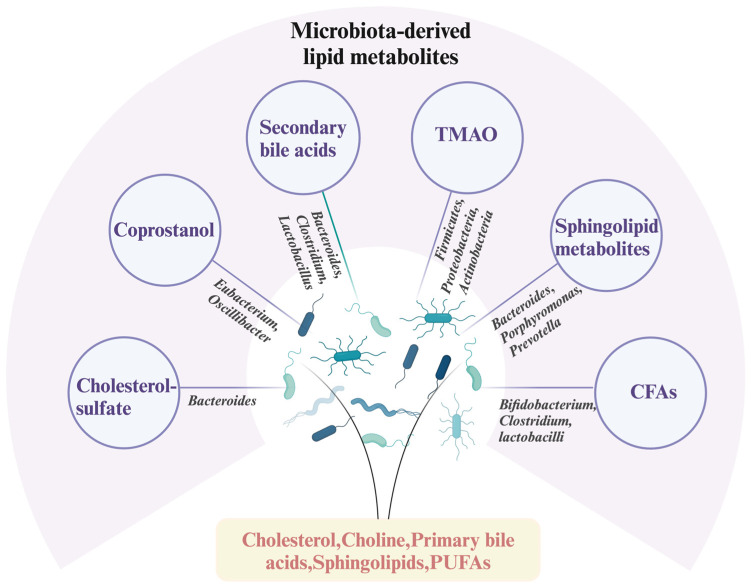
Gut-microbiota-derived lipid metabolites and their corresponding bacteria. TMAO: trimethylamino oxide; CFAs: conjugated fatty acids. (Created by Biorender.com).

The mammalian gastrointestinal system is colonized by a complex microbial ecosystem comprising hundreds to thousands of bacterial taxa that participate in host metabolic processes through nutrient breakdown, modification, and detoxification. These gut microbes not only regulate dietary lipid uptake but also have the ability to synthesize lipids. Therefore, we further discuss the biosynthesis and transformation of several major lipids by bacteria (Figure 1) and their modulatory impacts on host immunological responses and metabolic health.

### 3.1. Bile Acids

#### 3.1.1. Bile Acid Synthesis and Enterohepatic Circulation

Circulating cholesterol, derived from either dietary intake or de novo synthesis in hepatocytes, serves as a key precursor molecule and substrate for the biosynthesis of various lipid-derived metabolites, including bile acids, steroid hormones, and vitamin D [33]. Primary bile acid synthesis from cholesterol in the liver proceeds through two alternative enzymatic pathways: the classical (or neutral) pathway, which relies on cholesterol-7α-hydroxylase (CYP7A1) to generate cholic acid (CA) and chenodeoxycholic acid (CDCA), and the alternative (or acidic) pathway, which utilizes sterol 27-hydroxylase (CYP27A1) to produce CDCA [34]. Free bile acids are further activated to bile acid CoA esters by bile acid CoA synthetase (BACS) and then conjugated with taurine or glycine by bile acid-CoA: amino acid N-acyltransferase (BAAT) before being secreted into bile [35]. In the ileum, conjugated bile acids undergo active uptake by enterocytes primarily through the apical sodium-dependent bile acid transporter (ASBT). Following absorption, these bile acids associate with ileal bile acid-binding protein (IBABP) for subsequent translocation to the basolateral membrane [36]. Hepatocytes receive approximately 95% of bile acids back through portal circulation, where they are reprocessed before being re-excreted into bile together with newly synthesized bile acid species. Postprandially, these bile acid molecules are released into the intestinal tract, completing the enterohepatic cycle [37]. A minor proportion (around 5%) of bile acids undergo microbial modification in the intestinal tract, resulting in secondary bile acid production and consequent alterations in bile acid pool dynamics [38].

#### 3.1.2. Microbial Bile Acid Deconjugation

The generation of secondary bile acids from primary bile acids involves a microbiota-dependent two-reaction cascade. First, conjugated bile acids enter the intestine and undergo deconjugation by microbial bile salt hydrolase (BSH) [39]. The enzyme BSH is phylogenetically widespread across diverse bacterial taxa, but significant variations exist in its structural organization, biochemical characteristics (including pH optimum and kinetic parameters), and substrate preference [40]. A comprehensive classification of BSH proteins was identified in human intestinal bacteria involving a total of 591 bacterial strains in 117 genera across 12 phyla, including Bacteroidetes, Actinobacteria, *Clostridium*, Firmicutes, and Proteobacteria [41]. Genomic analyses of gut microbiota have revealed numerous homologous sequences encoding putative BSH enzymes, revealing species- or genus-specific differences in the organization and regulation [40]. The BSH protein encoded by CBAH-1 in *Clostridium perfringens* genome exhibits significant differences in protein size and amino acid composition compared to purified BSH from other *C. perfringens* strains. The observed activity (86% of wild-type) strongly suggests that multiple BSH gene variants may be present in *C. perfringens* [42,43]. De et al. (1995) [44] found that the deconjugation rate of glycodeoxycholic acid (GDCA) was significantly higher than taurodeoxycholic acid (TDCA) in *Lactiplantibacillus plantarum*. In contrast to wild-type controls, BSH-inactivated mutant strains demonstrated pH- and concentration-dependent susceptibility to GDCA. However, no such effect was observed for TDCA. The preferential binding of BSH to glycine-conjugated bile salts likely reflects the relative abundance of glycine conjugates over taurine conjugates in human bile acids (typically 3:1) [45].

#### 3.1.3. Bile Acid 7α-Dehydroxylation and the bai Operon in Gut Bacteria

Through 7α-dehydroxylation, the gut microbiota metabolizes deconjugated bile acids into secondary bile acids. Specifically, CA undergoes transformation to deoxycholic acid (DCA), while CDCA is modified into lithocholic acid (LCA). These bioconversions are regulated by the bile-acid-inducible (bai) operon, which is highly conserved in *Clostridium scindens* and *Clostridium hylemonae* strains [46]. A previous study proposed that *Eubacterium* might exhibit 7α-dehydroxylation activity. However, the species having the bai operon in *Eubacterium* were subsequently reclassified into the *Clostridium* [47]. Metagenomic sequencing analysis had revealed that the bai operon is mainly distributed in Firmicutes, particularly in the Lachnospiraceae and Ruminococcaceae [48,49]. Within the bai operon, baiG encodes a proton-dependent transporter facilitating primary bile acids import into cells [50]. ATP-dependent CoA ligation is mediated by baiB gene, while ATP-independent CoA transfer is encoded by baiF and baiK genes [51]. baiA catalyzes the oxidation of the 3-hydroxy group, and baiCD encodes NADH, a flavin-dependent oxidoreductase that introduces a C4=C5 bond in 7α-hydroxy, 3-dehydro-bile acids. The baiH gene encodes an enzyme homologous targeting 7β-hydroxy, 3-dehydro-bile acids [52]. The rate-determining reaction in this pathway, bile acid 7α-dehydration, is facilitated by the baiE protein, and baiI gene is proposed to encode 7β-dehydratase [53,54].

#### 3.1.4. Microbial Transformation of Bile Acids: Hydroxyl Isomerization and Oxidation

**Figure 2:**
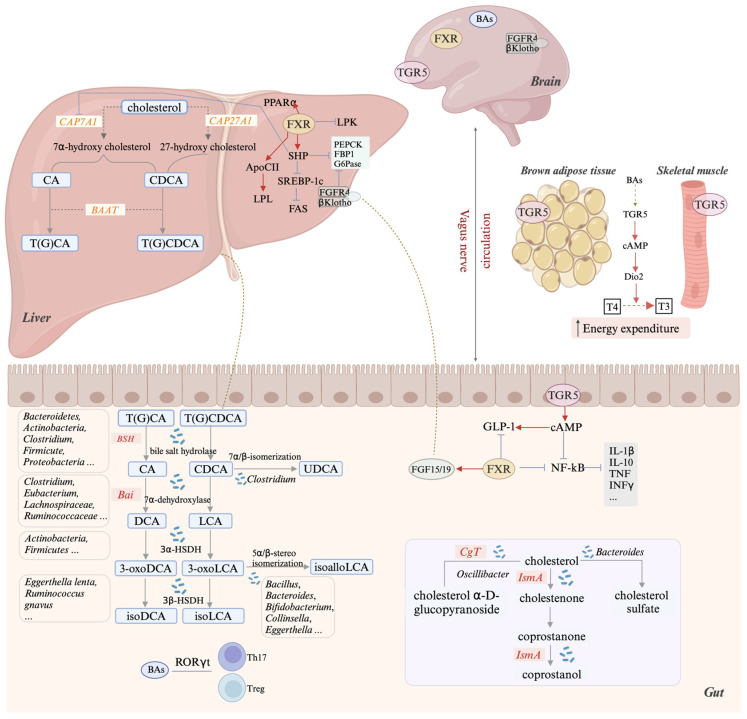
Gut microbiota, bile acids, and metabolic regulation. Hepatocytes synthesize primary bile acids from cholesterol through two distinct metabolic routes. Gut microbiota modifies primary bile acids into secondary bile acids through deconjugation (BSH), 7α-dehydroxylation (bai operon), and isomerization. The bacterial taxa involved in these processes are shown in boxes. Bile acids regulate host metabolism via FXR and TGR5 receptors. (Created by Biorender.com).

Microbiota can further enrich the bile acid pool through oxidation and isomerization of hydroxyl groups. Ursodeoxycholic acid (UDCA) demonstrates differential classification across species, being a primary bile acid in murine models but categorized as a secondary bile acid in humans, which can be generated from CDCA via 7α/β-isomerization by *Clostridium* [55]. The hydroxyl groups of bile acids can undergo oxidative isomerization, resulting in the conversion of DCA to 3-oxoDCA and LCA to 3-oxoLCA. This transformation is mediated by gut bacteria expressing 3α-hydroxysteroid dehydrogenase (3α-HSDH), primarily from Actinobacteria and Firmicutes [56]. Moreover, *Eggerthella lenta* and *Ruminococcus gnavus* catalyze the 3β-hydroxyl oxidation process, generating isoDCA and isoLCA [56]. Additionally, gut bacteria can perform 5α/β-stereo isomerization at the C5 position of bile acids. According to Li et al. (2021), the enzymatic conversion of 3-oxoLCA to isoalloLCA was observed in 16 species of bacteria distributed across 11 genera, including *Bacteroides*, *Bacillus*, *Bifidobacterium*, *Collinsella*, *Eggerthella*, and so on [57] (Figure 2).

#### 3.1.5. Bile Acid Receptors and Key Functions

The enterohepatic cycling of bile acids enables efficient breakdown and assimilation of dietary fats. Acting as chemical messengers, they play regulatory roles in lipid homeostasis and the maintenance of internal environment by activating multiple cellular receptors. Bile acid signaling is mediated through two distinct receptor families: nuclear receptors and membrane-bound receptors. The nuclear receptor family includes farnesoid X receptor (FXR), vitamin D receptor (VDR), pregnane X receptor (PXR), and constitutive androstane receptor (CAR), while the membrane-bound receptors comprise G protein-coupled bile acid receptor 1 (TGR5/GPBAR1) and sphingosine-1-phosphate receptor 2 (S1PR2) [58,59]. These receptors are widely distributed across various tissues, including, but not limited to, hepatic, intestinal, cerebral, adipose, and skeletal muscle, where they regulate diverse physiological processes. Among these receptors, FXR and TGR5 have been extensively studied.

FXR is a central regulator of bile acid synthesis, transport, and detoxification. Beyond these canonical roles, FXR critically modulates glucose and lipid metabolism through tissue-specific mechanisms. The bile acid-FXR signaling pathway in the liver negatively regulates bile acid synthesis through SHP-1-mediated inhibition of liver receptor homolog-1 (LRH-1), a key transcriptional activator of the CYP7A1 gene [60]. In the intestine, FXR activation stimulates the production and secretion of fibroblast growth factor 15 (FGF15; human ortholog FGF19), which circulates to the liver, binds to fibroblast growth factor receptor 4 (FGFR4), and inhibits *CYP7A1* expression via JNK pathway activation [61]. Together, these hepatic and intestinal FXR-dependent mechanisms tightly regulate bile acid homeostasis. Studies have demonstrated that FXR-knockout mice exhibit significantly elevated plasma and hepatic triglyceride levels [62]. Mechanistically, FXR—activated by endogenous bile acids—downregulates hepatic lipogenesis in an SHP-dependent manner by suppressing the expression of SREBP-1c and its target gene fatty acid synthase (FAS) [63]. The carbohydrate-responsive element-binding protein (ChREBP) plays a pivotal role in mediating the conversion of carbohydrates to lipids. Importantly, bile-acid-activated FXR has been shown to downregulate ChREBP expression, thereby suppressing de novo lipogenesis [64]. Furthermore, FXR exerts dual regulatory effects on lipid homeostasis by modulating peroxisome proliferator-activated receptor α (PPAR-α), a nuclear receptor that governs lipoprotein metabolism and fatty acid oxidation. Through this regulatory axis, FXR activation enhances fatty acid oxidative metabolism while concomitantly reducing lipid accumulation in hepatocytes [65]. Activation of hepatic FXR leads to reduced levels of phosphorylated JNK (p-JNK), which subsequently upregulates the expression of hepatocyte nuclear factor 4α (HNF4α) and scavenger receptor class B type 1 (SR-B1). This coordinated molecular cascade ultimately enhances high-density lipoprotein cholesterol (HDL-C) clearance from circulation [66]. Collectively, FXR emerges as a master regulator of lipid metabolism, coordinately controlling lipogenesis, fatty acid oxidation, and cholesterol transport, thereby maintaining metabolic homeostasis. In glucose metabolism, bile acids employ the FXR-SHP-dependent pathway to interact with nuclear transcription factors at the promoter regions of gluconeogenic genes (e.g., PEPCK, G6Pase), thereby suppressing their expression [67]. Furthermore, FXR directly binds to the promoter of liver pyruvate kinase (LPK), a key glycolytic enzyme, to inhibit its transcription. This suppression of glycolysis promotes glycogen storage, contributing to the hypoglycemic effect [64].

TGR5 can modulate energy homeostasis and glucose metabolism through mechanisms such as increasing insulin secretion [60]. According to research by Watanabe et al. [68], bile-acid-supplemented feeding in mice could boost energy expenditure in both brown adipose tissue and skeletal muscle. This thermogenic effect was mediated through the BAs-TGR5-cAMP-signaling axis, which upregulates type 2 iodothyronine deiodinase (Dio2) expression, thereby enhancing the conversion of thyroxine (T4) to active triiodothyronine (T3) and elevating oxygen consumption rates. Clinically, diabetic patients treated with high-dose TGR5 agonists exhibited significantly elevated glucagon-like peptide-1 (GLP-1) levels and improved glycemic control [69]. Notably, Hyocholic acids (HCAs) simultaneously activate the TGR5 receptor while inhibiting FXR. By acting on intestinal enteroendocrine L-cells, HCAs increase intracellular cAMP levels, enhance GLP-1 secretion, and effectively reduce blood glucose levels [61].

Bile acids serve as important immunoregulatory molecules in mammalian systems. The immunoregulatory effects of primary (CA, CDCA) and secondary (DCA) bile acids are mediated through receptor-dependent signaling in innate immunity [70]. For instance, DCA-TGR5 interaction significantly suppressed pro-inflammatory cytokine production (IL-1β, IL-6, IL-12p70, and TNF) in mice [71]. In neuroinflammatory models, TUDCA treatment exerted potent anti-inflammatory effects by activating TGR5, which suppressed microglial synthesis of inflammatory cytokines, including IFN-γ and TNF-α [72]. Experimental evidence from TGR5-deficient and TGR5-overexpressing mouse models revealed that TGR5-mediated cAMP induction in macrophages leads to potent anti-inflammatory effects through suppression of the NF-κB signaling cascade [73]. Patients with ulcerative colitis exhibit elevated NF-κB-dependent cytokine production and severe intestinal inflammation, which correlates significantly with reduced FXR expression [74]. Analysis of peritumoral liver tissue in hepatocellular carcinoma patients revealed positive associations between CDCA and both CXCL16 expression and NKT cell infiltration, contrasting with negative correlations observed for glycolithocholate [75]. Bile acid metabolites serve as immunomodulators in adaptive immunity, directly influencing the T cells. Studies in mice have demonstrated that feeding specific bile acid mixtures, such as CA/CDCA/UDCA and LCA/3-oxoLCA, affects RORγ-Tregs levels through activation of the VDR [76]. Hang et al. (2019) found that 3-oxoLCA inhibited Th17 differentiation by directly binding to the RORγt and inhibiting transcriptional potential in mice. Another LCA derivative, isoalloLCA, has been shown to promote Treg cell differentiation through a mechanism closely linking to excessive mitochondrial ROS accumulation and enhanced H3K27 acetylation at the FoxP3 promoter [77].

### 3.2. Microbial Conversion of Intestinal Cholesterol

Cholesterol is primarily absorbed in the small intestine. Unabsorbed cholesterol enters the colon, where it is converted into coprostanol by gut microbiota [78] (Figure 2). As a cis-isomer derivative of cholesterol, coprostanol is poorly bioavailable and primarily excreted in the stool, contributing to the lowering of cholesterol in the body [79]. The microbial coprostanol formation pathways diverge at the initial enzymatic step—either direct Δ^5^-bond hydrogenation or 4-cholelesten-3-one (cholestenone) intermediates formation—with isotopic tracing proving the latter pathway’s hydrogen transfer mechanics [80,81]. Thus, it is currently considered that bacteria first oxidize cholesterol to cholestenone, then reduce the double bond to produce coprostanone, and finally transform coprostanone to coprostanol in the gut [81]. The pathway initiation is catalyzed by either of two multifunctional enzymes: ChOx or 3β-HSD, both exhibiting consecutive enzymatic activities required for the sequential reactions [82]. Current evidence suggests distinct microbial strategies for cholesterol metabolism under different oxygen conditions. ChOx activity has been reported in aerobic microbial cultures [83], but its involvement in gut luminal cholesterol transformation appears unlikely due to the anerobic environment. This is supported by genomic analyses revealing no ChOx homologs in extensive human gut metagenomic datasets encompassing >3000 global fecal samples [84]. The aforementioned study, however, identified a functionally characterized 3β-HSD in *E. coprostanoligenes* ATCC 51222. It has been named *IsmA*, “intestinal steroid metabolism A”, and relies on NADP to exhibit oxygen-independent activity, representing an evolutionarily adapted mechanism for anerobic sterol metabolism in the gut ecosystem [84]. This enzyme was essential for the initial and terminal reactions in coprostanol generation, and bacteria carrying the *IsmA* gene were strongly related to reduced fecal and serum total cholesterol levels in a human cohort [84]. Researchers first isolated the cholesterol-reducing bacterium from rat intestines, tentatively classified as *Eubacterium* [85]. Comparative analyses reveal diminished coprostanol excretion in antibiotic-exposed subjects across species, contrasting with its undetectable status in axenic rodents and neonatal humans [86,87,88,89]. The abundance of *IsmA*-encoding bacterial species is significantly associated with gut health. Individuals with Crohn’s disease had decreased abundance of *IsmA*-encoding bacterial species in their guts compared to those without inflammatory conditions [78]. A recent study found that bacterial species from *Oscillibacter* metabolized cholesterol and contributed to the reduction in cholesterol and cardiovascular disease risk. These *Oscillibacter* species took up cholesterol and metabolized it into cholestenone, glycosylated cholesterol, and hydroxycholesterol through various pathways [90]. In addition to encoding the conserved *IsmA* enzyme, *Oscillibacter* species possess cholesterol-α-glucosyltransferase (CgT), which catalyzes the formation of glycosylated cholesterol derivatives (e.g., cholesterol α-D-glucopyranoside) through glucose conjugation [90]. This biotransformation increases cholesterol polarity, enhancing its fecal excretion. Interestingly, a low-carbohydrate, high-fat (LCHF) diet enhances microbial cholesterol-to-coprostanol conversion capacity in low converters, yet without significant effects on serum cholesterol levels. However, metabolically healthy lean individuals with high baseline conversion rates demonstrate increased LDL-cholesterol (LDL-C) levels following LCHF dietary intervention. These findings suggest that while microbial cholesterol conversion may reduce intestinal cholesterol, its systemic effects depend critically on both host metabolic status and dietary composition [91].

Additionally, it has been reported that *Bacteroides* spp. participate in the sulfation of sterol metabolites, with the enzyme BT_0416 in *Bacteroides thetaiotaomicron* being identified as the key catalyst responsible for converting cholesterol to cholesterol sulfate, thereby affecting serum cholesterol levels [92]. Cholesterol sulfate exhibits multiple biological functions. Studies have documented its involvement in cholesterol biosynthesis regulation, modulation of coagulation factors, and sperm maturation processes [93]. Particularly in cutaneous biology, cholesterol sulfate serves as an essential signaling molecule that orchestrates keratinocyte differentiation and stratum corneum development, critically maintaining epidermal barrier integrity [94]. Additionally, cholesterol sulfate promotes β-cell proliferation, protects against diabetic damage, and preserves function by maintaining GLUT2 expression and mitochondrial integrity.

These collective findings underscore gut microbiota in maintaining host cholesterol balance. Gut microbiota may modify the sterol backbone to generate more cholesterol derivatives through various biochemical pathways. However, the biological functions of microbial enzyme-derived cholesterol metabolites have not yet been fully elucidated and need to be further investigated.

### 3.3. Trimethylamine Oxide (TMAO)

#### 3.3.1. Microbial Conversion of Choline to Trimethylamine (TMA)

Choline, phosphatidylcholine, and L-carnitine derived from the diet or synthesized in vivo can be metabolized by gut microbiota. Some bacteria may utilize choline as a carbon and nitrogen source, and specific bacterial species containing trimethylamine lyase (TMA-lyase) can convert choline into trimethylamine (TMA). Following intestinal absorption, TMA enters the portal venous system and undergoes hepatic oxidation to TMAO, primarily mediated by the FMO3 isoform of flavin monooxygenases (FMO) [95]. TMA-lyase is a multi-enzyme complex composed of three distinct enzyme systems: cntA/B, cutC/D, and yeaW/X. CntA/B, comprising an oxygenase (CntA) and a reductase (CntB), was identified in *Acinetobacter calcoaceticus*, and *Serratia marcescens* could catabolize choline to TMA [96,97,98]. CutC, a specific glycyl radical enzyme activated by CutD, was initially identified in an anerobic sulfate-reducing bacterium *Desulfovibrio desulfuricans* [99,100]. YeaW/X has the similar protein sequence with cntA/B and mainly utilizes γ-butyrobetaine as a substrate for TMA biosynthesis [101,102]. Falony et al. (2015) compiled a catalog of gut bacteria with TMA-producing potential, and identified 102 reference genomes covering 36 related bacterial species predominantly categorized as Firmicutes, Proteobacteria, and Actinobacteria by using public genome databases [103]. A more comprehensive taxonomic analysis by Cai et al. (2022) using metagenomic sequencing data revealed 216 species harboring phylogenetically analogous segments of cntA/B, yeaW/X, and/or cutC/D. These bacterial species belonged to 102 genera, with *Lachnoclostridium* being the most abundant genus carrying the cutC gene. Further in vitro and in vivo experiments in mice confirmed that *L. saccharolyticum WM1* and choline administration promoted the development of atherosclerosis [104].

#### 3.3.2. TMAO as a Microbial Metabolite Linking Gut Microbiota to Cardiac Risk

The TMA-TMAO pathway is a newly identified mechanism by which choline and other methylamine-containing substances contribute to cardiovascular diseases through interactions with gut microbiota. Using metabolome data, TMAO has been established as an independent predictor of cardiac diseases, including heart failure, myocardial infarction, and stroke [105]. Fecal microbial transfer from high and low TMAO-producing mice into atherosclerosis-prone mice revealed that an upregulating abundance of TMA-producing bacterial species accelerated aortic injury [106]. A close relationship has been demonstrated in a mouse model between the changes in the abundance of gut bacteria related to TMAO generation and myocardial infarction. Oral administration of vancomycin in mice decreased serum leptin concentrations, reduced infarct size, and partially improved postischemic cardiac function. This effect was comparable to that obtained in *Lactobacillus plantarum* administration [107]. Therefore, modulating intestinal microbial communities to attenuate TMAO generation should be another promising therapeutic strategy for cardiovascular diseases.

### 3.4. Sphingolipid Metabolites

#### 3.4.1. De Novo Biosynthesis and Dietary Conversion of Sphingolipid Metabolites by Gut Microbiota

Sphingolipids represent a biologically active lipid class involved in numerous physiological functions, including cellular communication, inflammatory responses, and cellular differentiation processes [108]. The metabolic pathways for sphingolipid generation are best understood in eukaryotes, but interestingly, some gut-resident anerobic bacteria from the Gram-negative group have also demonstrated this biosynthetic function, including *Bacteroides*, *Porphyromonas*, and *Prevotella* [109]. Among these, species in *Bacteroides* are the most important sphingolipid-producing bacteria, synthesizing both odd-chain and even-chain sphingosine [110,111]. Serine palmitoyltransferase (SPT) initiates the first step in the de novo biosynthesis of sphingolipids [112]. Studies have shown that SPT is essential for Bacteroidetes persistence and stress adaptation in the gut. In *Bacteroides fragilis* and *Porphyromonas gingivalis*, SPT gene knockout or inhibition of SPT homologs render cells less resistant to oxidative stress [113]. Sphingolipids are important components of the cell membrane of Bacteroidetes and provide a variety of potential immunological active metabolites in the gut [114]. Bacteroidetes species could be colonized successfully in the stressful intestinal environment by a unique sphingolipid-mediated process and showed the benefit effect on the host [115]. Recently, sphingolipids have been used for the classification of bacterial taxonomies. A study on the sphingolipid composition of several Bacteroidetes species using thin-layer chromatography (TLC) and infrared spectroscopy revealed considerable heterogeneity in sphingolipid compositions among different species, whereas strains within the same species had similar sphingolipid profiles. Ceramide phosphorylethanolamine (CPE) and ceramide phosphoglycerol (CPG) have been detected in *B. ovatus*, *B. fragilis*, *B. uniformis*, *B. eggerthii*, *B. thetaiotaomicron*, and *B. stercoris* but not in *B. merdae*, *B. distasonis*, and *B. vulgates* [116].

In addition, diets can provide a substantial number of sphingolipids, which can be utilized by gut bacteria for biotransformation. During early infancy, humans are first exposed to exogenous sphingolipids through breast milk, followed by dietary sources, such as dairy products, meat, and eggs [117]. To precisely track the metabolic flux of dietary sphinganine and characterize microbial features involved in sphingolipid metabolism, Lee et al. (2021) developed the bioorthogonal labeling method (Bioorthogonal labeling-Sort-Seq-Spec, BOSSS) and identified a subset of bacterial species capable of efficiently uptaking dietary sphingolipids, including Bacteroidetes, which showed the ability to synthesize sphingolipids from scratch [118]. However, a study on early childhood cohorts revealed that *Bifidobacterium* can degrade complex sphingolipids despite lacking the genes encoding sphingolipid biosynthetic enzymes [119]. The high prevalence of Bacteroidetes and *Bifidobacterium* in gut microbial communities suggests an upregulation of sphingolipid biosynthesis and metabolic pathways in early life, which should serve as a predictive marker of health.

#### 3.4.2. Microbial Sphingolipids Modulate Host Immunity and Metabolism

Bacterial–host interactions mediated by sphingolipids play an essential role in regulating immune. Gut microbiota can increase sphingolipid levels, which in turn strengthen the intestinal barrier and reduce bacterial endotoxin exposure [120]. Microbial reconstitution of germ-free mice with sphingolipid-deficient bacteria has been shown to induce intestinal inflammation and alter the host ceramide pool [121]. Previous studies have demonstrated that *Bacteroides fragilis* has the potential to produce α-galactosylceramide, which attaches to the antigen-presenting molecule CD1d, modulates the quantity and functionality of intestinal natural killer T (NKT) cells, and influences the progression of colitis in mouse models [122]. In addition, sphingolipids produced by *Porphyromonas gingivalis* can be transported to host cells via outer-membrane vesicles (OMVs) and reduce inflammatory responses [123]. Using imaging tracking technology, researchers observed that sphingolipid metabolites, specifically homoserine-containing dihydroceramides (DHCers) that were derived from *Bacteroides thetaiotomicron*, could be effectively transferred to the colonic and hepatic tissues in mice. Further RNA-sequencing analysis revealed that HepG2 hepatocytes treated with homoserine-DHCers exhibited significant enrichment of differential expressed genes participating in oxidative phosphorylation, fatty acid metabolism, and glycolysis pathways, indicating an enhanced respiratory activity in hepatocytes [124]. Oral administration of sphingolipids extracted from *Acetobacter malorum* was efficiently absorbed in mice and subsequently metabolized in the liver into various sphingolipid metabolites [125]. These sphingolipid metabolites can even be identified in distal organs, such as the brain, indicating their ability to enter host cells for sphingolipid homeostasis [114].

### 3.5. Metabolism of Polyunsaturated Fatty Acids (PUFAs) by Gut Microbiota

Gut microbiota modulates host lipid metabolism by biotransforming dietary polyunsaturated fatty acids before they are utilized by the host. PUFAs saturation is a common metabolic pattern of PUFA metabolism by gut microbiota. Free PUFAs are converted into free saturated fatty acids with less toxicity to reduce their inhibitory effect on bacterial growth. This process reflects the detoxifying activity of anerobic bacteria, such as *lactobacilli* [126]. Microbial PUFA metabolism can produce various specific fatty acids, including conjugated and trans-isomeric forms. This condition was confirmed in mice, which showed that the levels of CLA isomers and non-conjugated derivatives in the intestinal contents of conventional mice were significantly increased compared to GF mice [127]. Certain microbial taxa, such as *Bifidobacterium*, *Lactobacillus*, and *Propionibacteria*, possess linoleate isomerase, which catalyzes the conversion of the omega-3/omega-6 PUFA precursors linoleic acid (LA) and α-linolenic acid (ALA) to CLA and conjugated α-linolenic acid (CLNA) [128,129]. For examples, potential enzymes involved in LA transformation were identified in the genome of *L. plantarum*, including the linoleate isomerase, acetoacetate decarboxylase, and oxidoreductase [130]. In a study screening thirty-six *Bifidobacterium* strains cultivated in vitro, six strains were identified with the ability to produce various CFA isomers, including four strains of *Bifidobacterium breve*, one strain of *Bifidobacterium bifidum*, and one strain of *Bifidobacterium pseudolongum* [131]. Hennessy et al. (2012) evaluated the linoleic acid isomerase activity of *Bifidobacterium* strains in CFA production and found that *Bifidobacterium breve* DPC6330, as the most important member, could transform 70% of LA to CLA and 90% of ALA to CLNA [128]. In addition, *Clostridium bifermentans* can hydrogenate arachidonic acid (AA) and eicosapentaenoic acid (EPA) to produce saturated fatty acid derivatives containing a trans double-bond configuration at the ω7 [132].

Conjugated fatty acid isomers have been revealed to confer anti-inflammatory activity, antitumor potential, and anti-obesity properties [133]. For example, c9,t11-CLA improves insulin sensitivity through enhanced glucose uptake and adipokine modulation (leptin reduction and adiponectin elevation) while exerting anti-atherogenic effects via PPARγ-mediated suppression of macrophage activity and plaque formation [134,135]. In parallel, the t9,t11 isomer demonstrates atheroprotective properties through LXRα activation, promoting cholesterol efflux and reducing inflammation [136]. These differential mechanisms highlight the isomer-specific therapeutic potential for managing metabolic syndrome and cardiovascular diseases. One potential mechanism by which PUFAs exert beneficial effects on host metabolism may be through the microbial production of CFAs.

## 4. Role of Probiotics in Modulating Lipid Metabolism

Gut microbiota can be broadly categorized into beneficial and harmful bacteria based on their physiological effects. Current guidelines define probiotics as viable microbes (primarily *Lactobacillus*, *Bifidobacterium*, and *Bacteroides*) that deliver measurable health benefits at adequate doses [137,138]. Accumulating evidence has demonstrated that probiotic supplementation modulates lipid metabolism. For instance, *Lactobacillus gasseri* suppressed adipose tissue expansion in murine models [139]. *Lactobacillus* strains overexpressing BSH attenuated hypercholesterolemia by reducing intestinal cholesterol absorption while stimulating its metabolic degradation in mice [140]. *Lactobacillus rhamnosus* altered gut microbiota composition and reduced total cholesterol and triacylglycerol levels in zebrafish larvae by modulating lipid-metabolism-related gene transcription [141]. These beneficial effects have been consistently replicated in human clinical studies. Substantial evidence demonstrates that milk fermented with lactic acid bacteria exerts hypolipidemic effects by suppressing hepatic cholesterol biosynthesis and reducing low-density lipoprotein levels [142,143]. A clinical study evaluating 43 volunteers demonstrated that prolonged enteral administration of selenium-enriched *Enterococcus faecium* M-74 probiotic strain significantly improved serum lipid parameters. After 56 weeks of supplementation, subjects showed a 12% reduction in total cholesterol concentrations [144]. Supplementation with *Bifidobacterium* and *Lactobacillus plantarum* strains modulated gut microbial function in atopic dermatitis patients by downregulating *Staphylococcus aureus*-associated colonization genes while upregulating steroid hormone biosynthesis pathways, ultimately improving immune responses [145].

Bacterial metabolites serve as critical cross-kingdom signaling molecules that enter the systemic circulation and regulate host lipid metabolism, particularly SCFAs and bile acids. SCFAs simultaneously enhance insulin sensitivity and exert tissue-specific metabolic regulation, including adipose lipid storage modulation, hepatic metabolic reprogramming, and skeletal muscle energy utilization optimization [146]. The portal vein delivers SCFAs not metabolized by intestinal epithelia to the liver, functioning as metabolic substrates for three key processes: glucose production, fat synthesis, and cholesterol generation [147]. Experimental evidence has demonstrated that exogenous butyrate induces metabolic remodeling in diet-induced obese mice through PPARγ-signaling suppression, transitioning cellular metabolism from lipid storage to oxidative pathways [148]. Gut microbiota regulates systemic lipid homeostasis via BSH-mediated bile acid deconjugation, a biochemical transformation that simultaneously enhances hepatic cholesterol catabolism through increased bile acid recycling and reduces intestinal lipid absorption by disrupting micelle formation [149].

So, understanding probiotic-mediated lipid regulation provides critical insights for developing novel therapies against metabolic disorders. Despite promising preclinical evidence, probiotic applications face key translational challenges: strain-specific efficacy variations, inadequate long-term clinical validation, and incomplete understanding of host–microbe interactions across diverse genetic background cohorts. Systematic screening of strains with effective lipid-modulating functions and synergistic bacterial combinations should be undertaken in the future. In order to establish clinical relevance, standardization, more comprehensive clinical trials employing randomization and control methodologies, along with sustained participant tracking are also needed.

## 5. The Effects of Microbial Lipid Metabolism on Pig Production

Lipid metabolism is critical for the efficiency and economic returns of pig production because it influences pig growth, development, meat quality, and health. Substantial studies have confirmed the crucial role of the gut bacteria in regulating lipid metabolism in swine (Table 1). A comparative analysis of the intestinal microbial communities in the duodenum, jejunum, and cecum of pigs with varying degrees of obesity revealed a significant enrichment of *Escherichia coli* across all intestinal segments in high-fatness pigs [150]. Further experiments in mice revealed that excessive fat consumption expedited the proliferation of *E. coli*, enhanced choline catabolism, and elevated circulating TMAO levels, which finally promoted fat accumulation in hosts [151]. However, appropriate choline supplementation could improve growth performance and mitigate intestinal inflammation in weaned piglets by modulating the gut microbiome and lowering triglyceride levels [152]. Feeding pigs with *Bifidobacterium breve* as a probiotic altered the composition of PUFAs. This alteration correlated with a decrease in pro-inflammatory mediators, notably TNF-α and IFN-γ [153]. *Clostridium butyricum* supplementation remodeled the gut microbiota composition and bile acid profile of intrauterine growth restriction (IUGR) piglets, accelerated fatty acid synthesis and oxidation, and improved piglet liver growth and morphology by reducing the biosynthesis of cholesterol and promoting cholesterol efflux to downregulate total cholesterol levels [154]. Transplantation of fecal microbiota from Laiwu pigs to DLY pigs significantly increased fatty acyls and glycerophospholipids in the gut and liver but reduced plasma triglycerides. This suggested that the microbiota might promote the conversion of triglycerides into fatty acyls. Additionally, this study suggested that the resistance ability of intestinal diseases in Chinese indigenous pig breeds might be associated with sphingolipids production by *Bacteroides* based on the significantly higher proportions of *Bacteroides* in Laiwu and FMT pigs along with the increased sphingolipid levels in the plasma of FMT pigs [155]. Bile acids have been considered as novel feed additives that efficiently enhance lipid absorption and improve growth performance in livestock and poultry [156,157]. Weaning piglets are particularly susceptible to intestinal barrier damage during feed transitions, leading to diarrhea and impaired growth rates. A previous study demonstrated that dietary supplementation with bile acids in weaned piglets modulated the gut microbiota composition, enhanced average daily gain, improved feed conversion efficiency, and alleviated the intestinal inflammation, ultimately contributing to better production performance [158]. Furthermore, supplementation of primary bile acid CDCA in feed elevated plasma levels of the growth-promoting nutrient GLP-2, which exerted protective effects on intestinal epithelial integrity in neonatal piglets and strengthened the protective functions of the intestinal mucosa [159]. Bile acid dysregulation in pregnant sows showed a detrimental effect on fetal growth, and it has been shown that the regulation of gut bacteria can improve the bile acid composition of sows, thereby improving their reproductive performance [160]. These findings indicate that modulation of intestinal microbial lipid metabolism may represent a viable strategy for enhancing swine production efficiency.

Currently, studies on microbiota-related lipid metabolites in swine are shifting toward functional investigations and mechanism elucidation. However, there are still many unknown aspects to explore in future studies. For example, how does the interaction between the gut microbiota and the host influence lipid metabolites in pigs? Which lipid metabolites in the pig gut impact production traits and what are the underlying mechanisms?

## 6. Conclusions and Perspectives

Gut bacteria exhibit comprehensive lipid metabolic functions, including both the synthesis of new lipid molecules and the enzymatic modification of host lipids and nutritional factors to produce diverse lipid metabolites. These lipid metabolites primarily play roles in the intestinal epithelium, circulation, and peripheral tissues, where they influence metabolic homeostasis, immune responses, and host physiology. With the development of lipidomic technologies, the quantification of lipid metabolites offers a straightforward read-out of the overall host–microbiota interaction. Thus, the lipid-centric study design is a valid approach for cohort-based research aimed at identifying microbial mediators of host health. The crosstalk between gut microbiota and lipid metabolism exhibits remarkable complex. It is crucial to elucidate the mechanisms underlying the relationships of lipid metabolites with gut bacteria, particularly in the context of host homeostasis and dynamic environmental changes, which poses several critical challenges.

Bacterial membrane lipids play essential roles in maintaining cell membrane integrity, regulating signal transduction, and mediating host–microbe interactions. However, their structural diversity and biological functions remain largely unknown. Certain specific lipid categories, such as cyclic and polar lipids, have been implicated in bacterial resistance and host immune regulations [161,162], yet their mechanisms of action remain unclear. Additionally, although bacteria and eukaryotes share the similar membrane lipid composition, they exhibit substantial differences in structural complexity and functional diversity of membrane lipids. Whether these differences reflect distinct evolutionary adaptations and biological requirements remains uncertain.

Another challenge for the studies on lipid metabolites is the difficulty in tracing their sources. In most cases, we do not know which lipid metabolites in the gut are specifically linked to gut bacteria. The presence of co-metabolism complicates the identification of the origin of certain lipid metabolites. Integrative analyses of metagenome and metabolome reveal the association between lipid metabolites and microbial taxa; however, future research should more focus on providing more conclusive evidence of causality. In addition, many “uncultured” or “unknown” intestinal microbes lack metagenomic annotations or reference genomes, resulting in incomplete characterization of the interactions between gut bacteria and lipid metabolites.

Furthermore, current technologies for measuring lipid metabolites, databases for data annotation, and algorithms for data analysis remain limited. Although significant advances in mass spectrometry (MS) and gas chromatography (GC), these technologies have still exhibited imperfection in sensitivity, resolution, and coverage, particularly when detecting low-abundance or structurally complex lipid metabolites. Additionally, lots of lipid metabolites cannot be annotated due to the limited databases. To address these challenges, it is necessary to develop more advanced lipidomic technologies, establish a more complete microbial lipidome profile, and utilize integrated multi-omics approaches, such as the combined analysis of single-cell sequencing, transcriptome, and culturomics. Based on this foundation, microbiota-related lipid metabolites should be symmetrically explored, and their functional roles of lipid metabolites in host physiology should be elucidated.

Moreover, it is crucial to explore the possible applications of lipid metabolites in animal production and human health. Several microbial lipid metabolites, such as CFAs, secondary bile acids, and sphingolipids, have demonstrated potential benefits in regulating host metabolism, enhancing immune function, and improving host health. In livestock production, optimizing the gut microbial composition to enhance lipid metabolism may improve feed efficiency, promote animal growth, and reduce disease susceptibility. Additionally, microbial interventions aimed at modulating lipid metabolism could enhance the quality of animal-derived products, such as increasing CFA content or optimizing fatty acid composition of meat. Therefore, the establishment of regulation methods for modulating gut microbiota and optimizing the lipid metabolite profiles should improve the efficiency of pig production and support pig health. More importantly, pigs are an important model for studying human diseases. The studies on pig lipid metabolism can provide the basic knowledge for developing therapies for obesity-related diseases.

## Figures and Tables

**Table 1:** The effects of microbial lipid metabolism on pig production.

Study and Treatment	Key Findings	Reference
Comparison of varying degrees of obesity in pigs	High-fat diet enriched *E. coli* in pigs, which may increase TMAO.	Yang et al. (2016) [150]
Choline supplementation for weaned piglets	Improved growth performance and reduced inflammation.	Qiu et al. (2021) [152]
Probiotic (*Bifidobacterium breve*)	Altered polyunsaturated fatty acid composition and reduced pro-inflammatory cytokines.	Wall et al. (2009) [153]
*Clostridium butyricum* in IUGR piglets	Enhanced fatty acid synthesis/oxidation, and reduced cholesterol levels.	Zhang et al. (2023) [154]
Fecal microbiota transplantation from Laiwu to DLY pigs	Increased fatty acyls and glycerophospholipids in gut/liver, reduced plasma triglycerides	Xie et al. (2022) [155]
CDCA supplementation in neonatal piglets	Increased GLP-2 and enhanced intestinal barrier integrity.	Jain et al. (2012) [159]
Bile acid regulation in sows	Enhanced fetal growth and reproductive performance.	Wu et al. (2021) [160]

## Data Availability

No new data were created or analyzed in this study.
